# What methods do reviews of normative ethics literature use for search, selection, analysis, and synthesis? In-depth results from a systematic review of reviews

**DOI:** 10.1186/s13643-017-0661-x

**Published:** 2017-12-19

**Authors:** Marcel Mertz, Daniel Strech, Hannes Kahrass

**Affiliations:** Institute of History, Ethics and Philosophy of Medicine, Hannover Medical School, Carl-Neuberg-Str. 1, D-30625 Hannover, Germany

**Keywords:** Systematic review, Literature review, Normative literature, Argument-based-literature, Empirical ethics, Bioethics, Evidence-based medicine

## Abstract

**Background:**

(Semi-)systematic approaches to finding, analysing, and synthesising ethics literature on medical topics are still in their infancy. However, our recent systematic review showed that the rate of publication of such (semi-)systematic reviews has increased in the last two decades. This is not only true for reviews of empirical ethics literature, but also for reviews of normative ethics literature. In the latter case, there is currently little in the way of standards and guidance available. Therefore, the methods and reporting strategies of such reviews vary greatly. The purpose of the follow-up study we present was to obtain deeper methodological insight into the ways reviews of normative literature are actually conducted and to analyse the methods used.

**Method:**

Our search in the PubMed, PhilPapers, and Google Scholar databases led to the identification of 183 reviews of ethics literature published between 1997 and 2015, of which 84 were identified as reviews of normative and mixed literature. Qualitative content analysis was used to extract and synthesise descriptions of search, selection, quality appraisal, analysis, and synthesis methods. We further assessed quantitatively how often certain methods (e.g. search strategies, data analysis procedures) were used by the reviews.

**Results:**

The overall reporting quality varies among the analysed reviews and was generally poor even for major criteria regarding the search and selection of literature. For example, only 24 (29%) used a PRISMA flowchart. Also, only 55 (66%) reviews mentioned the information unit they sought to extract, and 12 (14%) stated an *ethical* approach as the theoretical basis for the analysis. Interpretable information on the synthesis method was given by 47 (60%); the most common methods applied were qualitative methods commonly used in social science research (83%).

**Conclusion:**

Reviews which fail to provide sufficient relevant information to readers have reduced methodological transparency regardless of actual methodological quality. In order to increase the internal validity (i.e. reproducibility) as well as the external validity (i.e. utility for the intended audience) of future reviews of normative literature, we suggest more accurate reporting regarding the goal of the review, the definition of the information unit, the ethical approach used, and technical aspects.

**Electronic supplementary material:**

The online version of this article (10.1186/s13643-017-0661-x) contains supplementary material, which is available to authorized users.

## Background

The number of publications of (semi-)systematic reviews for finding and synthesising ethics literature has increased in the last two decades [1]. This is not only true for reviews that search and synthesise ethically relevant empirical literature, but also for reviews of normative literature. Similarly, the scholarly debate on the opportunities and limitations of (systematic) reviews in ethics has expanded. This is particularly true for reviews of normative literature [2–4], which is also reflected in philosophy, one of the disciplines most concerned with ethics [5].

Since established standards for the conduct of especially *systematic* reviews of normative literature are still lacking, in a recent study [1], we assessed how reviews of normative literature report on their methods for search, selection (including quality appraisal), analysis, and synthesis of the literature and also compared the reporting to an (adapted) version of criteria related to PRSIMA statements [6]. Proposals for systematic review methods are rare and rather vague when it comes to analysis and synthesis of ethics literature [7, 8]. The more comprehensive and technical manuals on review methodology [9], guideline development [10–12], and Health Technology Assessments [13–16] also lack explanation of how to search, analyse, and synthesise relevant information from the ethics literature in a systematic and transparent manner (cf. [17]).

Search methods and, to a certain degree, selection methods for (systematic) reviews of normative literature can use tried and tested methods for general (systematic) reviews with the important difference that in ethics, books and book chapters can have much relevance, which has to be reflected in a search, selection, and also quality appraisal strategy. However, the experience of researchers undertaking such reviews is often that the desired normative literature is not easy to find [18]. Defining inclusion and exclusion criteria can also be harder than in, for example, reviews of clinical studies. As far as analysis and synthesis methods are concerned, it is becoming clear that qualitative research approaches are much more relevant in this kind of review than in “traditional” systematic reviews, such as those of clinical studies [19], but there is a lack of meta-research to show which methods can be employed in reviews of normative literature and how.

Therefore, in order to obtain deeper insight into how the reviews searched for selected, analysed, and synthesised normative information, in this paper, we take a closer look at the specific steps and processes used and the methodological information reported. Thereby, our analysis aimed both to identify common (“uniform”) steps or strategies in the search, selection, analysis, and synthesis methods of reviews on normative literature, and at the same time to capture existing variance in these methods. Both are needed to understand the current state of the art of such reviews and to provide the basis for future methodological improvement.

## Methods

### Study registration and PRISMA checklist

No review protocol was published beforehand, and the review was not registered (e.g. with PROSPERO). The description of methods follows the PRISMA statement [6] as far as applicable to this kind of review (see Additional file 1 for PRISMA checklist).

### Search and selection

**Fig. 1:**
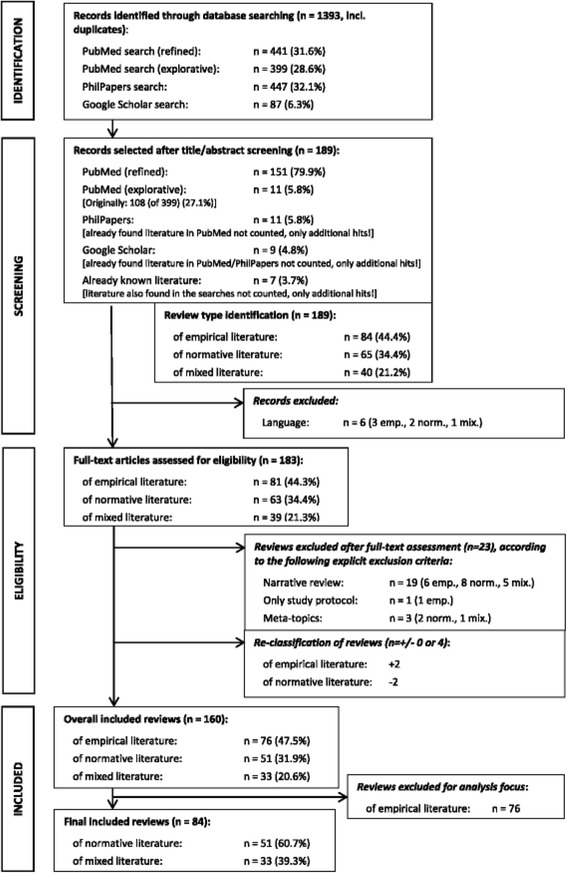
PRISMA flow diagram (originally published in [1]; see for further details also [1])

The search and selection method we used to identify (semi-)systematic reviews of ethics literature is described in more detail in [1]. In summary, our review of reviews was based on two PubMed searches, with additional searches in PhilPapers and Google Scholar, in April 2015. The searches produced 1393 hits, of which 189 were deemed relevant based on title or abstract and 183 after full-text screening. (See Fig. 1). Only articles which focused on the normative and “mixed” (empirical *and* normative) literature (*n* = 84) were analysed, leaving aside reviews that solely focused on empirical literature (*n* = 99). However, we also included “empty reviews” which explicitly stated that they found or included 0 hits if their research question matched our inclusion criteria. Because of language barriers, only articles in English, German, or French were considered for in-depth analysis.

### Analysis

#### Development of the coding matrix

We used a combined deductive and inductive strategy to construct categories [20, 21] for a *coding matrix* (coding frame). The deductive component encompassed the overarching categories related to the different basic methodical steps of a review, i.e. search, selection, quality appraisal, analysis, and synthesis, relative to the PRISMA statement [6].

To develop fine-grained subcategories, an inductive strategy was employed. Therefore, introduction and method sections (or equivalent textual parts) of a purposive sample of *n* = 20 articles were read. Descriptions of search, selection, quality appraisal, analysis, and synthesis methods were extracted. First, two researchers (MM, HK) each analysed five reviews of normative and five of mixed literature, discussed their suggested revisions, and finally agreed on refinements of the preliminary coding matrix. This was done in two consecutive rounds including ten more articles. Then, the plausibility of the categories developed was checked by the third researcher (DS). A further random sample of 10 articles was then examined to identify potentially overlooked issues. Because no subcategory was added, categorical saturation had been reached and the coding matrix was deemed final.

#### Main analysis of the individual reviews

To analyse methods applied by the reviews, qualitative content analysis (QCA) [20, 21] was used. The analysis was independently conducted for all included reviews by two researchers (MM, HK). Usually, only explicit statements about search, selection, quality appraisal, analysis, and/or synthesis were considered as reported. However, when both researchers independently judged that a certain method was used implicitly in a review, this was also coded accordingly. The analysis employed closed (yes/no) and open answer modes. Any disagreement was discussed with the third researcher (DS) and agreement sought. We did not send the extracted data to the authors of the reviews to check if they agreed with our analysis mainly due to time constraints. Also, an earlier pilot study trying to do such a “member check” showed a rather low response rate [22], which casts at least some doubts on the success of a similar strategy in this review.

### Synthesis

The synthesis method for the information extracted was mainly quantitative, e.g. how often the date/period of the search was stated (closed answer mode). In categories with open modes of data, the individual answers were counted (e.g. about which databases were used in a review) or subsumed under a broader description (e.g. approaches to becoming acquainted with the text). For the latter, we defined new synthesis categories corresponding either to what the authors of the reviews explicitly stated or to what we, on the basis of our experience and expertise in ethics and empirical methodology, found the most appropriate interpretation of what was said about the methods or procedures.

## Results

In our systematic review, we identified 84 reviews published between 1997 and 2015 in 65 different journals [1]. We included *semi-systematic* reviews that had at least an identifiable description of a reproducible literature search (search and selection) as well as (full) systematic reviews that further explicitly or implicitly reported on analysis and synthesis. When comparing the reporting to an (adapted) version of criteria related to PRISMA statements [6], only a small fraction of the included reviews fulfilled all criteria (for search 8%, for selection 21%, for analysis 8%, and for synthesis 11%) [1].

### Methods used for search, selection, and quality appraisal

**Fig. 2:**
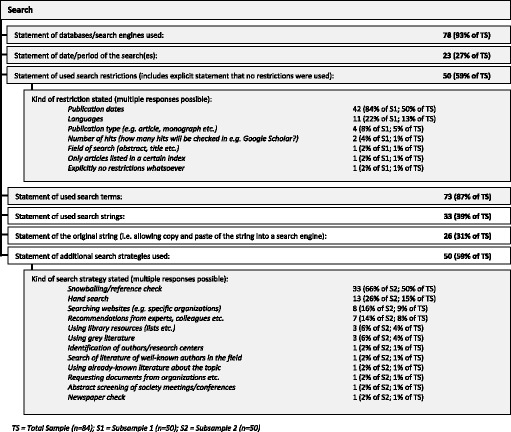
Search methods used in the reviews

Most reviews stated the databases or search engines they used (*n* = 78, 93%) and the search terms (*n* = 73, 87%). Search strings were mentioned to a lesser extent (*n* = 33, 39%); the statement was not counted if the information about the search string did not allow replication of the search. If it was replicable, we differentiated between sufficient information (e.g. Boolean operators for search terms) and the statement of a “copy-paste” search string (“original” search string). Of these 33 reviews, 26 (78%) presented a search string that allows for copying and pasting it directly into the database or search engine. (See Fig. 2.)

**Fig. 3:**
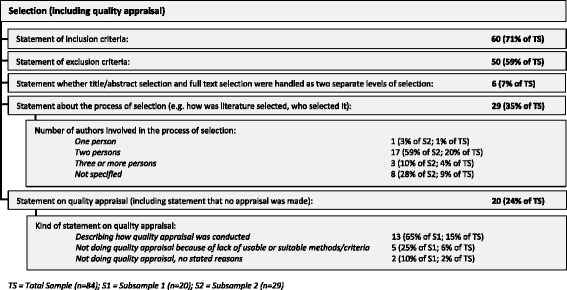
Selection methods used in the reviews

Search restrictions were mentioned by 50 reviews (59%), with publication date restrictions (*n* = 42, 50%) and language restrictions (*n* = 11, 13%) mentioned most often. Even though exclusion criteria were stated less often (*n* = 50, 59%) than inclusion criteria (*n* = 60, 71%), only 14 (17%) of the reviews stated neither inclusion nor exclusion criteria. Hardly any reviews made clear whether there was a difference regarding the selection criteria and/or selection procedure between selection at the title/abstract level and at the full-text level (*n* = 6, 7%). (See Figs. 2 and 3).

Of the 20 reviews (24%) that addressed the issue of quality appraisal of the literature they included, one in four (*n* = 5, 25%) explicitly wrote that they disregarded quality appraisal procedures and also stated an explicit reason for this by mentioning that there are no usable or suitable methods or criteria for a quality appraisal of normative literature. There were also few reviews (*n* = 2) that stated without further explanation that they did not apply a quality appraisal, making it difficult to understand the rationale for forgoing quality appraisal. The remaining reviews (*n* = 13) mentioned how they conducted the quality appraisal. (See Fig. 3.)

**Fig. 4:**
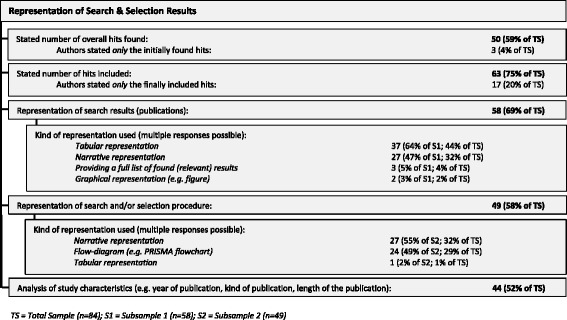
Representation of search and selection results used in the reviews

Finally, most reviews stated the number of results retrieved (*n* = 50, 59%) and included (*n* = 63, 75%). This number reflects the mentioned number, irrespective of whether the numbers included duplicates and further search strategies. About a third (*n* = 24, 29%) of the reviews used a PRISMA flowchart to represent the search and selection procedure. (See Fig. 4.)

#### Databases/search engines used

**Fig. 5:**
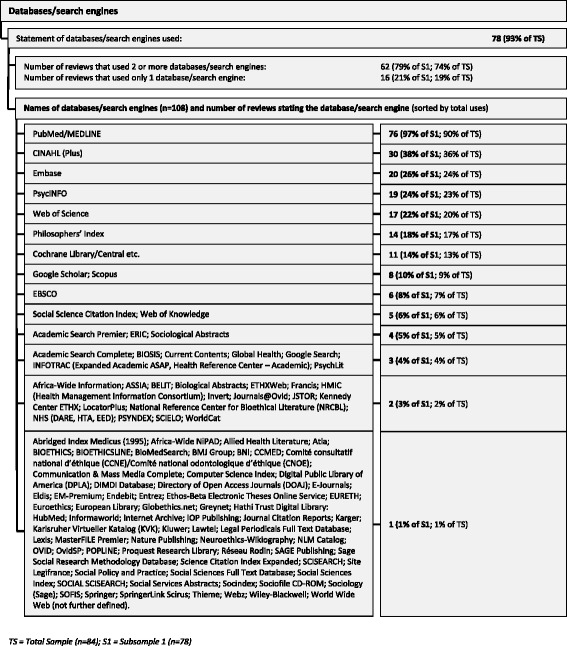
Databases/search engines used in the reviews

Of the 84 reviews, 78 (93%) stated which databases or search engines they used for the search (multiple responses possible). Overall, there were 108 different databases or search engines used, though they were not equally popular. Of the 78 reviews, nearly all used at least *PubMed*/*MEDLINE* (*n* = 76, 97%). Other databases or search engines often mentioned were *CINAHL* (*n* = 30, 38%), *EMBASE* (*n* = 20, 26%), *PsycINFO* (*n* = 19, 24%), and *Web of Science* (*n* = 17, 22%). Many databases and search engines were mentioned only once (*n* = 68, 87%). Furthermore, most reviews used at least two databases or search engines (*n* = 62, 74% of all reviews, 79% of the reviews that stated databases/search engines). Of the 16 reviews that relied on one database or search engine, 14 (88%) used PubMed/MEDLINE (see Fig. 5).

### Methods used for analysis and synthesis

#### Analysis

**Fig. 6:**
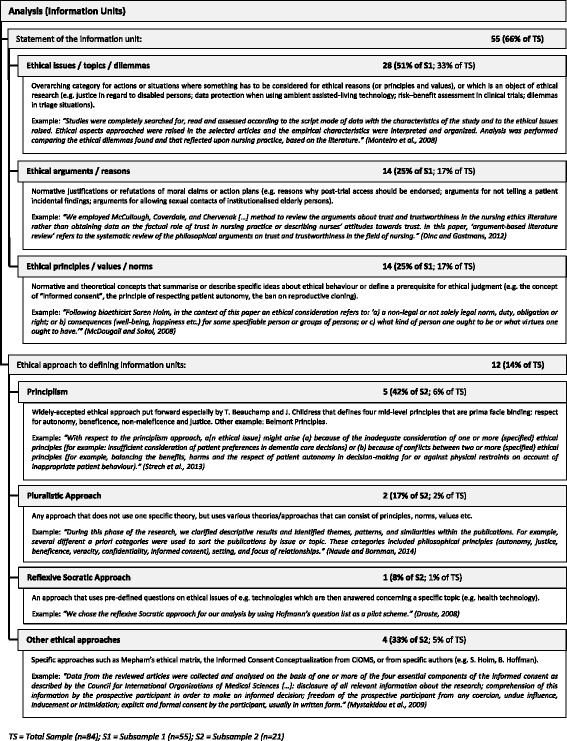
Analysis methods used in the reviews (information units)

In our sample of 84 reviews, 55 (66%) mentioned what kind of normative information unit they sought to extract from the material and synthesise thereafter. Twenty-one (25%) stated the theoretical approach they used to define information units (e.g. methods of qualitative analysis such as qualitative content analysis or grounded theory, definitions of philosophical arguments, or sociological theories regarding organisational justice), and of these 21, 12 (57%) gave sufficient information regarding the use of an *ethical* approach (e.g. ethical theory, framework, principles etc.). (See Fig. 6).

For the information unit we differentiated between (1) ethical issues/topics/dilemmas, (2) ethical arguments/reasons, and (3) ethical principles/values/norms. Of the 55 reviews stating the information unit, half extracted ethical issues, topics or dilemmas as information units (*n* = 28, 51%), a quarter extracted ethical arguments or reasons (*n* = 14, 25%), and a further quarter ethical principles, values, or norms (*n* = 14, 25%). Of the 12 reviews mentioning the ethical approach, “principlism” was dominant (*n* = 5, 42%). (See Fig. 6.)

**Fig. 7:**
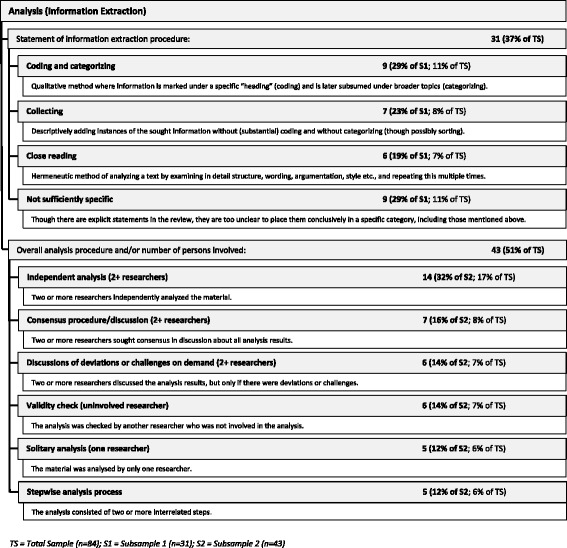
Analysis methods used in the reviews (information extraction)

Thirty-one (37%) reviews mentioned the procedure used to extract the information. Most used a “coding and categorising” approach (*n* = 9, 29%); in another nine reviews (29%), we found their statements to be too unclear to place them conclusively in one of the three procedure types that we were able to identify in our analysis. (See Fig. 7.) On the other hand, 43 (51%) said something about the overall analysis procedure, e.g. how many researchers were involved and whether there were, for example, consensus rounds between researchers if they disagreed with an analysis result. Of these 43, 33 (77%) seemed to involve more than one researcher in the analysis process; 5 (12%) stated that the analysis was done only by one researcher (of which 1 review was written by a sole author, which means that the entire analysis in the other 4 reviews, although written by two or more authors, was performed nonetheless by one researcher). (See Fig. 7.)

### Synthesis

**Fig. 8:**
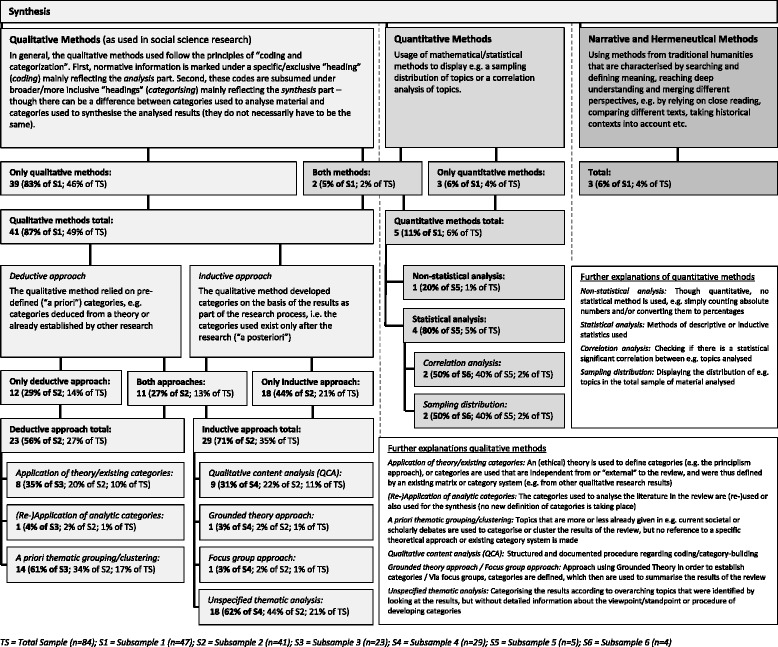
Synthesis methods used in the reviews

Of our sample, 47 (60%) reviews gave interpretable information about the way they synthesised the analysed material. Of these 47, 39 (83%) used qualitative methods (as mainly understood in empirical social science research), 3 (6%) used quantitative methods, 2 (5%) used both qualitative and quantitative methods, and 3 (6%) reported some kind of narrative or hermeneutic methods (as understood in humanities traditions). (See Fig. 8.)

Regarding qualitative methods (*n* = 41), 12 (29%) employed a purely deductive approach (a priori defined categories, e.g. based on a theory or framework), 18 (44%) a purely inductive approach (a posteriori built categories), and 11 (27%) combined deductive and inductive approaches.

Deductive approaches mostly relied on existing thematic groupings of the debate or literature (*n* = 14, 34% of the 41 reviews using a qualitative approach), while some involved a theory or another existing category system (*n* = 8, 20%). One review re-applied the categories from the analysis process for the synthesis (2%). Inductive approaches were often not interpretable regarding a specific approach; these we categorised under “Unspecified Thematic Analysis” (*n* = 18, 44%). The other reviews with inductive approaches used qualitative content analysis (*n* = 9, 22%), grounded theory (*n* = 1, 2%), or a focus group (*n* = 1, 2%). Here again, we took implicit as well as explicit information into account. (See Fig. 8.)

Reviews that used a quantitative approach (*n* = 5) to synthesis mostly relied on statistical analysis (*n* = 4, 80% of the 5 reviews using a quantitative approach), be it a correlation analysis or an analysis of the distribution of the topics (both *n* = 2, 40%). One review (20%) used a quantitative approach that relied not on a specific statistical analysis, but was mere counting. (See Fig. 8.)

## Discussion

In this paper, based on a systematic review of reviews, we present detailed findings on how 84 reviews of normative or “mixed” literature report their methods for the search, selection, analysis, and synthesis of relevant information.

### Applied search and selection methods

The search and selection methods used correspond to the items in the PRISMA guidelines [1, 6]. Therefore, there are not many qualitative differences from methods of more established kinds of (semi-)systematic reviews. It is known, though, that there are some practical complications in both searching [18] and selecting normative literature. These include the interdisciplinarity of the field and the resultant variation in terminology, publication standards, and journals [18], which might be reflected in the use of many databases (*n* = 108) in the sample of reviews in our meta-review.

Possibly because of this, only a few of the reviews used one database or search engine (21%). One might argue that using more databases and search engines leads to better results. However, it is unclear whether this hypothesis holds true for reviews of normative literature. On the basis of our analysis, we cannot make any statement about the effectiveness of using several databases and search engines. Better reporting of the attribution of included results and duplicate search results from different sources would be necessary in order to prove this hypothesis.

PubMed/MEDLINE was by far the most common database (97%). On the basis of our data, however, we cannot elucidate the reasons for its prevalence. It might be that PubMed/MEDLINE is deemed a relevant and fruitful database not only for biomedical topics, but also for bioethical topics as well. It could also be that PubMed/MEDLINE is regarded as a “standard” database that is commonly used in systematic reviews related to health care and is thus preferred. Another explanation, namely that other databases/search engines are not widely known, seems unsubstantiated in the face of the large number mentioned (see Fig. 5).

Given the possible problems in searching literature and given the humanities tradition of ethics as a “book” discipline, it is interesting that additional search strategies are not so widespread. About 60% of the reviews stated that they used such strategies, with “hand search” (e.g. manually checking monographs, collections) being mentioned by 26%; most mentioned was “snowballing”/“reference check” (66%). Though there is no evidence currently that hand search can improve the amount or quality of normative information gathering, it seems plausible to assume that in fields such as ethics—which traditionally rely on book publications which are harder (or impossible) to find with some of the common databases/search engines—such additional search strategies would yield greater returns than in, e.g., systematic reviews of clinical interventions.

### Applied analysis and synthesis methods

There is a clearly discernible tendency to favour qualitative approaches in both analysis and synthesis in our sample of reviews of normative literature (with “qualitative approaches”, we refer to an overarching category that encompasses both qualitative (social science) methods and narrative or hermeneutic methods insofar as they are both non-quantitative methods). In the 31 reviews that reported information extraction (analysis) procedures, “coding and categorising” dominates (29%); but “close reading” was almost as popular (19%). “Collecting” (23%) could also be understood as a quantitative way of information extraction; most often, though, our observations indicated that it too was used in a qualitative way.

Furthermore, of the 47 reviews that stated or described their synthesis methods, 44 (94%) used qualitative methods (*n* = 41, 87%) or narrative/hermeneutic methods (*n* = 3, 6%). Quantitative methods were much less represented (*n* = 5, 11%) (see Fig. 8). This tendency towards the use of qualitative approaches for reviews of normative information is supported by the findings of Tricco et al., who reviewed emerging knowledge synthesis methods for complex interventions in health care [19].

When using qualitative methods, the authors of the analysed reviews separately applied inductive or a posteriori categorisation (71%), and deductive or a priori categorisation (56%), or used a mixed approach (27%). When using deductive categorisation, a third (35%) relied on an (ethical) theory or existing category system, which is interesting given the fact that ethical theories in particular offer ample opportunities to define a priori categories for synthesising normative information. The presence of thematic analysis that is not specified in more detail (62%) and qualitative content analysis (31%) makes clear, however, that at the same time inductive strategies might often be better suited for this task. Arguably, this also depends on the goals of the review and the type of information unit sought.

Further research is needed to assess whether the synthesis of information units such as ethical issues works better with the use (additionally) of deductive/a priori categories, or if in general, the openness of inductive strategies is essential for finding suitable synthesis categories. Arguably, this depends on synthesis objectives. Here, one might differentiate two broad objectives: first, reproducibility (securing “internal validity”); second, “ease of use” and/or utility for the intended audience (securing “external validity”). “Ease of use”/utility in this regard might also be understood as “ecological validity”, that is the applicability of the results to the real-world setting of practitioners working in the health care system.

The overall tendency towards qualitative approaches in form of text analysis and synthesis seems natural, as normative information is extracted from texts and is itself textual or conceptual information. This might be a reason why it is not always explicitly stated. But as there is some variety regarding methods, in particular for the synthesis (see above), it is still important information for readers of such reviews.

### Reporting quality and transparency

The variance mentioned above regarding qualitative approaches shows that not only the methods used varied among the reviews in our sample, but also the *reporting* of the methods varied greatly—even for the well-established standardised search and selection part of the reviews (see Figs. 2 and 3). The more topic-specific analysis and synthesis parts of the reviews did not fare much better (see results in Figs. 6, 7, and 8).

However, it is important to note that the lack of a statement does not necessarily imply methodological negligence. Sometimes, not having stated something or, more particularly, not having *done* something, can make perfect sense given the aim of a review, the time available, and the limits of publication (e.g. article length). For example, in a review with a purely descriptive purpose of developing a comprehensive spectrum of ethical issues at stake in a specific health care situation, bypassing quality appraisal of the included literature can be appropriate [23, 24]. However, to demonstrate awareness of this key element of a traditional systematic review, a possible minimal standard could be to say why no quality appraisal was done. Hereto, our review reveals insufficient reporting, as only 24% included any information about quality appraisal.

Some information essential for the reproduction of systematic reviews was lacking in many reports. Although databases and search terms were often reported, we found that 61% of reviews did not give enough information on the search string to reproduce the search, and 59% reported on search restrictions. Even more important, a definition of the used information unit was given by only 25% of authors. In such cases, we would argue that the reviews do not provide enough relevant information to the reader, which therefore reduces their methodological transparency (e.g. impeding an external assessment of their “internal validity”), regardless of the actual quality of the review conducted.

Further, there is information we would not describe as key to transparency which would nevertheless benefit readers. For example, our review indicated that only three reviews (4%) provided a separate full list of the included publications, besides mentioning them in the normal references of the article. Such a list can be most useful, as it makes it easier to identify the literature included. Because of practical limitations such as article length, authors might consider providing further information in an online supplement.

### Further developments towards best practice standards

Improved reporting in ethics reviews could increase overall methodological transparency, and thereby methodological quality, formally and informally. This is because an author who has to describe the methods used in meaningful detail will probably reflect more upon these methods and their use. Furthermore, a reader can be sensitised to questions of quality by reading a detailed report of methods. Finally, more explicit reporting enables formal analysis of how methods are used and how they could be improved.

To this end, having first identified different methods to conduct such reviews, a next step would be to define quality criteria or develop a reporting guideline for systematic reviews of normative literature. The fact that some reviews did not explicitly describe the methods used also shows that there might be a lack of relevant information for untrained *readers* of such reviews, who are unable to interpret the text to reconstruct the missing information. This can be a further motivation for reporting guidelines, as they can not only orient researchers conducting ethics reviews, but also the often interdisciplinary readership of this sort of review. The Q-SEA instrument [25], though developed for the quality assessment of ethical analyses in the context of Health Technology Assessment (HTA), could be understood in part as such a guideline, as it describes key issues of (systematic) literature search and inclusion/exclusion criteria, and sees the subsequent reporting of these issues as key to the process quality of an ethical analysis in HTA.

We would argue that when reporting on reviews of normative literature more transparency is particularly important regarding the following methods/parts of a review in order to increase “internal validity” as well as “external validity”:

- *Goals of the review*: goals definitely play a role in the way the synthesis is framed for the intended audience (academics, HTA professionals, guideline development groups etc.). An ethics review can be descriptive (e.g. “What are the issues?”) as well as prescriptive (“What should one do?”). Both are valid goals for such reviews, but have to be stated clearly as they can influence the methodological aspects of a review.
- *Information unit definition:* the normative information analysed and synthesised in the reviews should be stated more explicitly, e.g. ethical issues, arguments or principles and values.
- *Ethical approach:* stating the theoretical approach (e.g. ethical theory, framework, set of principles or values) used for defining the information unit to analyse and synthesise improves the understanding of these steps and clarifies some of the inevitable normative underpinnings.
- *Technical aspects:* systematic reviews should strive to describe all information needed to reproduce all steps performed to produce the reported results. Furthermore, the overall use of PRISMA flowcharts could be improved.

### Limitations

The analysis procedure was dependent on our background knowledge and our interpretation of the texts. We tried to counter the subjectivity of this approach by double assessment (MM, HK) and by a critical review of the intermediate results (DS). However, this does not give absolute protection against mistakes, or guarantee that our value judgements are shared by every reader. Therefore, there might be some leeway regarding the (synthesis) categories themselves as well as the placing of the methods used in reviews in specific (synthesis) categories. Also, because of time constraints, we did not check for inter-rater reliability by having two (or more) researchers analyse the same literature. We did this for some articles at the beginning, with the aim not to improve the quality of the data, but to identify problems in applying the analysis matrix, to refine category descriptions, and to formulate (further) decision rules where necessary.

## Conclusions

Together with our first paper [1], this paper is, to our knowledge, the first analysis of the state-of-the-art of (systematic) reviews of normative and mixed ethics literature on medical topics. It provides an in-depth view of the search, selection, quality appraisal, analysis, and synthesis methods used by such reviews. This information could be used to inform future reviews (e.g. what aspects of the method to report, what databases to use, approaches to use for synthesis, and so on), to write methodologies on conducting reviews in ethics, or to develop reporting guidelines for such reviews. The results of our study indicate that pursuit of the latter two goals especially would be worthwhile for improving the quality of conduct, transparency of reporting, and feasibility of evaluating reviews of normative literature.

## Additional file

Additional file 1: PRISMA checklist. (DOC 67 kb)

## Abbreviations

HTA: Health Technology Assessment
